# Increasing incidence of rotator cuff surgery: A nationwide registry study in Chile

**DOI:** 10.1186/s12891-021-04938-7

**Published:** 2021-12-20

**Authors:** Catalina Vidal, María Jesús Lira, Rodrigo de Marinis, Rodrigo Liendo, Julio J. Contreras

**Affiliations:** 1 Department of Orthopedics and Trauma, Pontifical Catholic University of Chile, Diagonal Paraguay #362, 8330077 Santiago, PC Chile; 2 Research Unit, Pontifical Catholic University of Chile, Diagonal Paraguay #362, 8330077 Santiago, PC Chile; 3 Shoulder and Elbow Unit, Pontifical Catholic University of Chile, Diagonal Paraguay #362, 8330077 Santiago, PC Chile; 4 Shoulder and Elbow Unit, Instituto Traumatológico, San Martin #771, 8340220 Santiago, PC Chile; 5 Department of Orthopedics and Trauma, Universidad de Chile, Independencia #1027, 8380453 Santiago, PC Chile

**Keywords:** Rotator Cuff Surgery, Nationwide Registry, Chile, Health insurance

## Abstract

**Background:**

The rotator cuff surgery (RCS) incidence is rising rapidly in North America, Europe, Asia, and Australia. Despite this, multiple factors limit patients’ access to surgery. In Latin America, barriers to orthopedic surgery have been largely ignored. The purpose of this study was to calculate the rate of RCS in Chile between 2008 and 2018, investigating possible associated factors to access such as age, sex, and the health insurance.

**Methods:**

An ecological study was carried out with nationwide data obtained from the Database of Hospital Discharges of the Department of Statistics. All Chilean inhabitants aged 25 years or more were included. We used the ICD-10 codes M751, M754, and S460. The annual incidence rate of surgeries and the incidence rate for the period studied per 100,000 inhabitants were calculated. Data were analyzed stratified by age, sex, year of study, and the health insurance. Negative binomial regression was used to compare rates. Statistical analyzes were performed with Stata v.14 software.

**Results:**

39,366 RCSs were performed, with a total rate for the period of 32.36 per 100,000 inhabitants. The annual rate of surgeries from 2008 to 2018 increased from 24.55 to 49.11 per 100,000/year. When adjusting for year, an annual increase in surgery rates of 8.19% (95% CI 6.7–9.6) and 101% growth between 2008 and 2018 (95% CI 90–109%, p < 0.001) was observed. When comparing the global rates according to the health insurance, the public system corresponds to 21.3 per 100,000 and the private system to 72 per 100,000, the latter being 3.4-times higher (95% CI 2.7–4.4; p < 0.001).

**Conclusion:**

RCS rates are increasing in Chile concordantly with previous reports of other western countries. The most important factor associated with RCS rate found was the patients’ health insurance, with higher rates observed for the private sector.

## Background

Shoulder pain is a frequent musculoskeletal complaint, affecting between 4.7% and 46.7% of the adult population each year [1]. Rotator cuff tears (RCT) are the leading cause of shoulder-related disability, especially in patients over 50 years [2–4]. Nonetheless, asymptomatic RCT also become more prevalent at older ages with up to 20% prevalence for patients in their sixties [5, 6]. It is estimated that around one third of patients with asymptomatic RCT will develop pain in the next 2 to 5 years, with higher pain rates related to tear enlargement [7]. Initial non-operative management is indicated in most cases of degenerative RCT with approximately 80% success rate reported [7]. If non-operative treatment fails, rotator cuff repair (RCR) may be effective in reducing pain and improving function [6, 8, 9]. Moreover, better long-term outcomes have been associated with rotator cuff healing after repair [10].

An increasing surgical volume is reported [2–4] probably due to population ageing. RCR is the most frequent shoulder surgery in the United States [11], and most of the patients that undergo surgery are in their fifth or sixth decade of life [3]. A cost-effectiveness analysis showed that, compared with non-operative treatment, the lifetime age-weighted mean total societal savings per patient from RCR is $13,771 [12]. The RCR incidence is rising rapidly in North America, Europe, Asia, and Australia [13–20]. Despite this, multiple factors, such as health care organization, culture, educational level, socioeconomic, and geographic factors, limit patients’ access to surgery [21]. In Latin America, lower rates of orthopaedic surgery are reported probably due to economic factors and limited access to health care in comparison with developed countries [22]. The rate of RCR in Brazil performed through the Brazilian Unified Health System increased from 0.83 to 2.81 per 100,000 between 2003 and 2015 yet remaining lower than developed countries [23].

Chile is a developing country, and its economy is known as one of the steadiest in the continent. In 2010, it became the first full member of the OECD in South America due to the recognition of its economic advances in the last decades, social development, and strong institutional restructuring. In Chile, public and private health insurances differ widely on their access to elective orthopedic surgery, with limited evidence being available. Historically, the private system has shorter waiting times, more health personnel, and better access to surgical opportunities.

Since 2005, to increase access to surgical procedures for the public health system, a new out of pocket spending (OOPS) approach has been allowed for rotator cuff surgery (RCS) and several other procedures [24]. Probably, the implementation of this strategy has increased the rate of RCS in patients from the public health system. Nevertheless, the rates of RCS in Chile are unknown to date.

The purpose of this study was to calculate the rate of RCS in Chile between 2008 and 2018 based on a nationwide registry, investigating possible associated factors to access such as age, sex, and the health insurance. We hypothesize that the rates of RCS have increased over the years and there are significant differences between private and public health insurance.

## Methods

### Study design and setting

An ecological study was carried out with nationwide data obtained from the Database of Hospital Discharges of the Department of Statistics (DEIS) of the Chilean Ministry of Health (MINSAL) between 2008 to 2018 (most current database). Founded in 1964, DEIS collects information on all hospitalizations in Chilean public and private care settings. These data are entered on a mandatory basis for all health centers around the country, considering both inpatient and outpatient care, and provides information on age, gender, health insurance (public or private), diagnosis, and year of surgery. This database is open access (https://deis.minsal.cl/).

### Population

Population data were obtained from the National Statistics Institute (INE) for each year. All Chilean inhabitants aged 25 years or older were included, considering that the number of surgeries in those under 25 years of age was low (5,468 surgeries). We used the ICD-10 codes M75.1 “Rotator cuff tear or rupture, not specified as traumatic”, M75.4 “Impingement syndrome of shoulder”, and S46.0 “Injury of muscle(s) and tendon(s) of the rotator cuff of shoulder” to identify patients who received surgical treatment for a rotator cuff disease (these codes include all patients who perform surgery for rotator cuff disease in Chile). These codes are used for both open and arthroscopic RCS. Therefore, this coding system did not allow us to differentiate between open and arthroscopic techniques. Also, concomitant procedures associated with RCS, such as acromioplasty, tenotomy, or tenodesis of the long head of the biceps tendon and resection of the distal end of the clavicle, do not have a specific code and are considered within the comprehensive management of the pathology.

### Statistical analysis

Descriptive statistical analyses were used to calculate the annual number of RCS in the Chilean population aged 25 or older. The annual incidence rate of surgeries and the incidence rate for the period studied per 100,000 inhabitants were calculated. Negative binomial univariate regression was used to compare age, sex, year of study, and the public or private health insurance, individually. Multivariate regression was used to compare differences between private versus public health insurance adjusted by age and sex, calculating the Incidence Rate Ratio (IRR). The model was based on the following equation: *E*[*Y*_*t*_] = exp {*β*_0_ + *β*_1_[Health Insurance] + *β*_2_[Year] + *β*_3_[Sex] }, where β0 is the intercept and β_1-3_ are coefficients for the data analyzed. The model that provides the best fit was selected. For the population data not available, the value was imputed by taking the mean of the data from the year before and the year after. Statistical analyzes were performed with Stata v.14 software.

### Ethics Committee

This project was approved by the scientific ethics committee of the Faculty of Medicine of the Pontificia Universidad Católica de Chile (ID: 16-196).

## Results

**Fig. 1:**
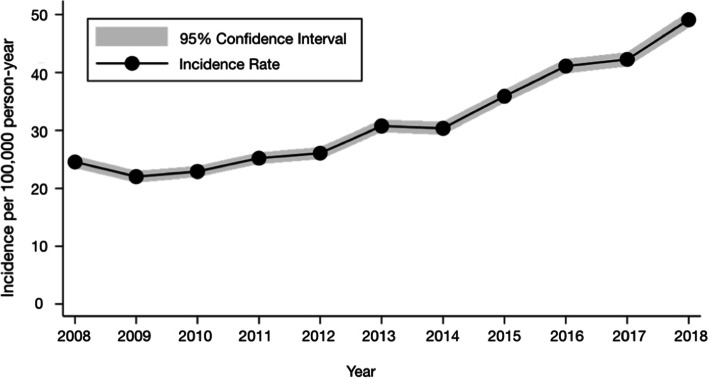
Incidence rate of rotator cuff surgery by year

During the period analyzed, 39,366 RCSs were performed, with a total rate for the period of 32.36 per 100,000 inhabitants. Female patients correspond to 54.26% (n = 21,360); 68% (n=21,135) and 15% (n=4745) were surgeries in the 45–64- and 65–74-year-old groups, respectively. The annual rate of surgeries from 2008 to 2018 increased from 24.55 to 49.11 per 100,000/year (Figure 1). When adjusting for year, an annual increase in RCSs of 8.19% (95% CI 6.7–9.6) and 101% growth between 2008 and 2018 (95% CI 90–109%, p < 0.001) was observed.

**Fig. 2:**
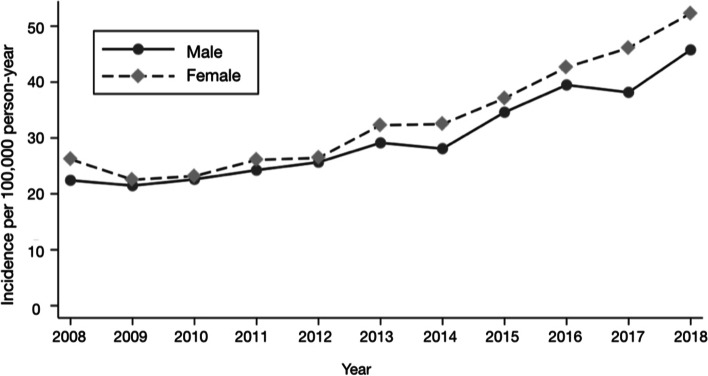
Incidence rate of rotator cuff surgery by sex

The increase in the rate of RCSs for female patients was significantly higher throughout the period compared to males (33 vs. 30 per 100,000 for the total period), with a difference of 10.7% in rates (p < 0.01) (Figure 2, Table 1).

**Table 1:** Comparison of incidence rate between groups.

Variables		IRR	Std. Err.	CI 95%	*p-value*
Incidence rates by year					
	*2008 (reference)* *2018*	1.08	0.01	1.07 - 1.09	<0.001
Incidence rates by sex					
	*Male (reference)* *Female*	1.11	0.01	1.09 - 1.13	<0.001
Incidence rates by age					
	*25-44 (reference)* *45-64*	6.71	0.11	6.49 - 6.95	<0.001
	*65-74*	5.89	0.13	5.65 - 6.14	<0.001
	*>75*	1.65	0.06	1.54 - 1.77	<0.001
Incidence rates by health insurance					
	*Public (reference)* *Private*	3.42	0.44	2.66 - 4.39	<0.001

Negative binomial regression was used for the individual analysis of each variable.

Abbreviations used IRR: incidence rate ratio, Std. Err: standard error, CI: confidence interval.

**Fig. 3:**
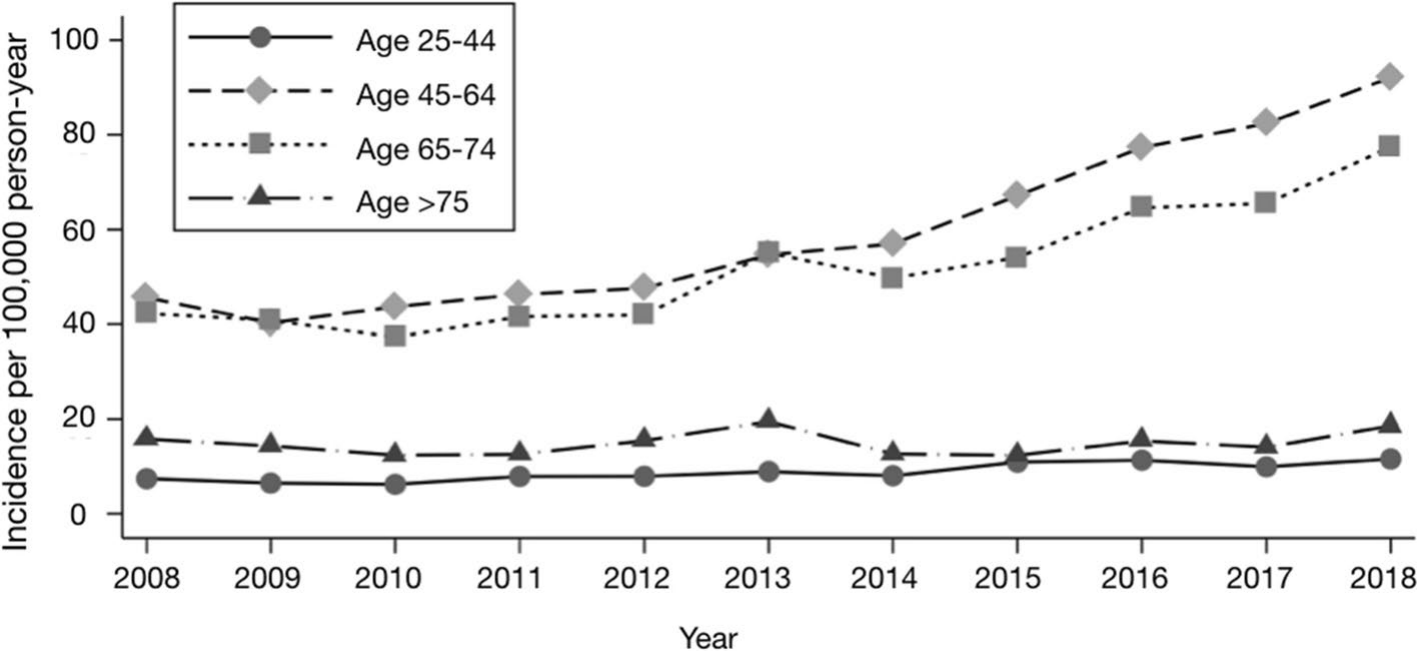
Incidence rate of rotator cuff surgery among patients in different age groups

The age ranges with the highest incidence of surgeries were the 45–64- and 65–74-year-old groups, with an overall rate of 61.6 and 53.2 per 100,000, respectively (compared to 8 per 100,000 in the group under 25 years and 14 per 100,000 in the group over 75 years) (Figure 3, Table 1).

**Fig. 4:**
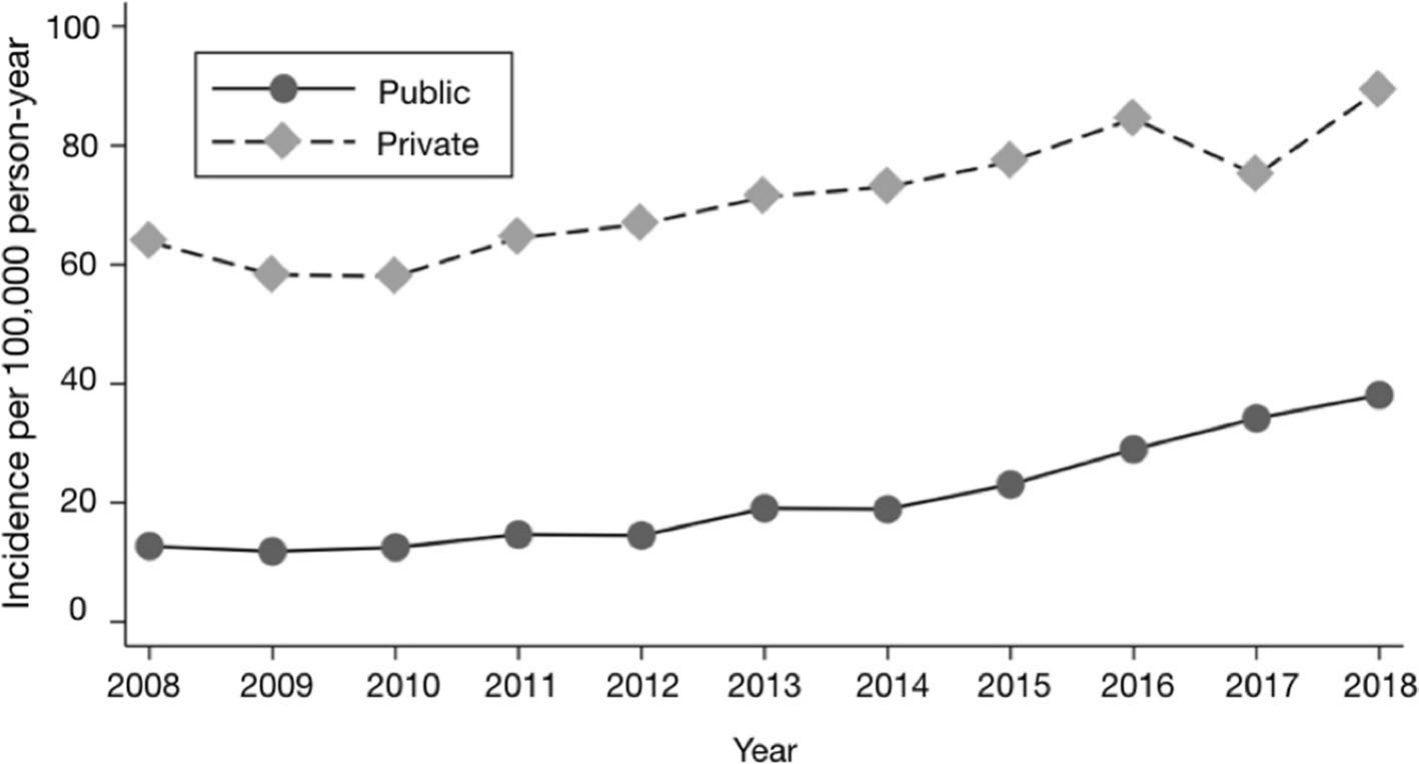
Incidence rate of rotator cuff surgery among patients in public and private health insurance

During the observed period, 19,564 surgeries were performed on patients enrolled in the public health insurance and 15,886 in the private system. However, when comparing the global rates according to the health insurance (Figure 4), the public system corresponds to 21.3 per 100,000 and the private system to 72 per 100,000, the latter being 3.4-times higher (95% CI 2.7–4.4; p < 0.001) (Table 1 and Table 2). In 2008, RCS rates in the private health insurance were 5 times higher than the public system. By 2018 this difference decreased to 2.34.

**Table 2:** Comparison of incidence rate between public and private health insurance

	Variables	*β*	IRR	Std. Err.	CI 95%	*p-value*
	*Health Insurance* *Public (reference)* Private (*)	1.56	4.79	0.26	4.29-5.34	<0.001
	Sex					
	*Male (reference)* *Female*	0.12	1.13	0.06	1.02-1.27	0.24
Age						
	*25-44 (reference)* *45-64* *65-74* >75	2.04 2.05 0.97	7.68 7.72 2.64	0.57 0.58 0.22	6.63-8.89 6.65-8.96 2.25-3.11	<0.001 <0.001 <0.001

(*) Age and sex adjusted multivariate analysis.

Abbreviations used IRR: incidence rate ratio, Std. Err: standard error, CI: confidence interval.

## Discussion

The main finding of this study is the increase in the rates of RCS in Chile during 2008 to 2018, in accordance with the literature published internationally, being the first national report on RCS rates.

The increase in the RCS rates has been greater in the female gender, the age group between 45 and 64 years, and with private health insurance. However, these are associations and not necessarily causal factors. There is likely a combination of factors such as ageing of the population, access to advanced imaging studies, technologic improvements in surgical instrumentation and increased number of trained surgeons explain this increase. Nevertheless, the lack of standardized criteria at national level to indicate RCS, lack of national clinical guidelines and specialized programs for the resolution of this pathology could favor a surgical over treatment of this pathology. The information obtained from the databases only reports the performance of the surgical procedure and not the details of the pathology (partial / complete tear, involved part of the rotator cuff, type of surgical procedure, clinical results) or the justification for the surgical indication.

During the 10-year study period, the calculated rate of RCS was 32.36 procedures for every 100,000 Chilean inhabitants over 25 years old; below the rate reported for European countries (Italy, 62.1 per 100,000 [2001–2014] [15], Finland, 44 to 131 per 100,000 [1998–2011] [20]); the United States (41 to 98 per 100,000 [1996–2006] [17]), and Korea (13.15 to 116.04 per 100,000 [2007–2015] [19]). Compared with RCS rates for Brazil (0.83 to 2.81 per 100,000 [2003–2015] [23]), we found a higher rate in a similar period. The causes of these differences were not evaluated in this study, but they are most likely multifactorial (socioeconomic factors, organization of healthcare systems, standardized criteria for surgical indication at national level, improvements in surgical technique). The organization of the healthcare systems is a relevant factor in the standardized resolution of this pathology. In Italy and Finland, the main source of financing is national and regional taxes, supplemented by copayments for pharmaceuticals and outpatient care [25]; furthermore, the presence of standardized clinical guidelines regulates the surgical indication and the use of healthcare resources [26].

RCS rates present statistically significant differences according to the variables analyzed. In Chile, there is a higher rate of surgery observed in women (male:female ratio 0.84:1) throughout the period, in contrast with the higher rates in men in other countries. In Finland, the male to female ratio is 1.7:1 [20], while in Korea and Italy is 1.02:1 [15, 19].

However, there are similarities in the age range, with the highest rate of RCS between 45 and 65 years [15]. In Korea, the mean age of patients who received RCS was 55.4 years (SD ± 10.8) [19]. The mean patient age at the time of operation in Finland increased from 55 (SD 9) years in 1998 to 56 (SD 10) years in 2011 [20]. In the US, it is precisely this age group (45–65 years) that presents the greatest increase in RCS (2 to 10 per 100,000 to 21 to 146 per 100,000), mainly with arthroscopic technique [17].

The main difference with respect to international reports is in relation to the health insurance. In 2014, 97.33% of RCSs were performed in Italian public hospitals and 2.67% in the private health insurance [15]. In 1998, 91% of rotator cuff repairs were performed in Finnish public hospitals and 9% in private hospitals, but in 2011, the corresponding percentages were 53% and 47% [20]. In Chile, the rate in the private health insurance is triple the rate in the public health insurance for RCS. This difference is possibly related the patient’s capability to cover costs and greater access to surgery compared with the public health insurance. The chilean healthcare system is significantly affected by economical segmentation with the minority of the population covered by private health insurance [27]. These increased rates could also be related to over diagnosis or over treatment in the private system. Furthermore, patients with private insurances are more likely to have better health literacy in contrast to persons not having a private health insurance [28]. The lower rates of RCS in the public sector could be explained by its shortage in coverage in terms of number of health care professionals per patient and concordantly the long waiting lists associated to this fact. Moreover, seek care in the private system without a proper coverage comes with a high out of pocket cost which limits public to private transfer of patients.

Nonetheless, the public system RCS rates increased at a higher pace than private RCS rates, observing a decrease in the difference at the end of the studied period (2018). In an attempt to explain this finding, we hypothesize that this may be due to: 1) an improvement in the public health insurance surgical capacity, 2) an increase in the state budget for the health sector, and/or 3) the implementation of new OOPS to access for RCS in the public health insurance.

Chilean citizens had a high level of OOPS and segmentation of private and public insurance schemes. The OOPS as a share of total health expenditure is 33% which is one of the highest among OECD countries (OECD average of 20%). The new OOPS payment or “Payment Associated to Diagnosis” (PAD) allows patients to pay an affordable, known, and fixed amount to access to packaged medical benefits depending on each diagnosis. This amount corresponds to 50% of the total amount received by the private institution that will provide health care (in this case RCS). The other 50% is covered by the state and is transferred directly to a private institution that must be affiliated to this system. As the amount paid per patient is fix, there are no subsequent modifications allowed in the total price independently from the number of implants used, bed days, medications, exams, and other supplies needed to carry out the procedure [24].

One of the main strengths of this study is the use of a large sample size based on a nationwide database of public information that must be compulsorily recorded in all surgeries of public and private institutions. Because the information is collected from all regions of Chile, the data are expected to be representative of the country. To our knowledge, this is the first study in Chile that reports RCS rates and analyzes its associated factors and differences with previously reported data from other countries.

One of the main limitations is the retrospective design, which can influence the recording and coding of the information. By including three different codes, it is expected to cover the greatest number of accurate diagnoses. However, we recognize that subjects may be excluded or incorrectly included we are unable to evaluate potential inaccuracies in the coding of the diagnoses. Nonetheless, we believe that our results highlight important trends in RCS.

## Conclusions

RCS rates are increasing in Chile specially for patients between 45 and 65 years old concordantly with previous reports of other western countries. Patients’ health insurance is associated with RCS rate, with higher rates observed for the private sector. The rate breach between public and private sector is diminishing over the observation period. Future studies may elucidate the associated factors that explain this difference and its trend over time.

## Data Availability

The datasets analyzed during the current study were derived from the following public domain resources: https://deis.minsal.cl/

## Abbreviations

RCS: Rotator cuff surgery
RCT: Rototar cuff tear
RCR: Rotator cuff repair
OECD: Organisation for Economic Co-operation and Development
OOPS: Out of pocket spending
DEIS: Database of Hospital Discharges of the Department of Statistics
MINSAL: Chilean Ministry of Health
INE: National Statistics Institute
PAD: Payment Associated to Diagnosis
